# Understanding the risk and protective factors associated with obesity amongst Libyan adults - a qualitative study

**DOI:** 10.1186/s12889-018-5411-z

**Published:** 2018-04-13

**Authors:** H. Lemamsha, C. Papadopoulos, G. Randhawa

**Affiliations:** 1 Faculty of Medical Sciences, University of Omar Al-Mukhtar, Al-Bayda Campus, Labraq Road, Al-Bayda, B1L12 Libya; 2 Institute for Health Research, University of Bedfordshire, Putteridge Bury Campus, Hitchin Road, Luton, LU2 8LE UK

**Keywords:** Obesity, Libya, Risk factors, Benghazi, Healthcare professionals, Community leaders

## Abstract

**Background:**

There are a range of multifaceted behavioural and societal factors that combine to contribute to the causes of obesity. However, it is not yet known how particularly countries’ cultural norms are contributing to the global obesity epidemic. Despite obesity reaching epidemic proportions in Libya, since the discovery of oil in 1959, there is a lack of information about obesity in Libyan adults. This study sought to explore the views of key informants about the risk and protective factors associated with obesity among Libyan men and women.

**Methods:**

A series of qualitative semi-structured interviews were conducted with Libyan healthcare professionals and community leaders.

**Results:**

Eleven main themes (risk and protective factors) were identified, specifically: socio-demographic and biological factors, socioeconomic status, unhealthy eating behaviours, knowledge about obesity, social-cultural influences, Libya’s healthcare facilities, physical activity and the effect of the neighbourhood environment, sedentary behaviour, Libyan food-subsidy policy, and suggestions for preventing and controlling obesity.

**Conclusions:**

Key recommendations are that an electronic health information system needs to be implemented and awareness about obesity and its causes and consequences needs to be raised among the public in order to dispel the many myths and misconceptions held by Libyans about obesity. The current political instability within Libya is contributing to a less-active lifestyle for the population due to security concerns and the impact of curfews. Our findings have implications for Libyan health policy and highlight the urgent need for action towards mitigating against the obesity epidemic in Libya.

## Background

Within the Libyan context, obesity has become a critical problem due to its prevalence amongst adults aged 20–74 years [1, 2]. Obesity has more than doubled, from a prevalence of approximately 12.6% in 1984 to 30.5% in 2009 [1–4]. The direct costs immediately associated with the cost spent in connection with the treatment of obesity-related co-morbidities in Libya in 2012 was estimated to be 1.3 billion Libyan Dinar per annum (approximately £638 million) which constituted 50–65% of Libya’s total health care budget [1, 5]. Ongoing conflicts in Libya among various radical militias have created an unstable political, social and economic environment. This unsafe environment is likely to be having an impact upon Libyans’ daily activities, for example, encouraging citizens to remain indoors for extended periods of time, consequently exacerbating the obesity epidemic.

Aspects of the Libyan culture may also be contributing to the obesity problem. For example, the perception that Islamic religion places constraints against women engaging in physical activity [6, 7]; inaccuracies in knowledge over what constitutes a healthy diet due to cultural habits (for example, the perception that consuming olive oil reduces cholesterol) [8]; fattening rituals for women before marriage [9]; and social gatherings in which guests are presented with large quantities of food and over-eating is encouraged [10, 11]. The role of culture is particularly relevant given that, according to Christakis and Fowler [12], obesity is ‘socially contagious’ and spreads from person to person in a social network.

A substantial body of scientific literature confirms that there is a lack of information about obesity in adults in North African Arab countries, including Libya [13–16]. In recent years, the second largest city in Libya, Benghazi, has experienced a rapid increase in chronic diseases that have been linked to obesity, leading to disability and premature death [17, 18]. Due to these gaps in the literature and the likely importance of cultural norms impacting upon obesity in the Libyan context, we set out to explore the views of Libyan healthcare professionals and community leaders about the risk and protective factors associated with obesity in Benghazi including the wider role of Libyan culture.

## Methods

### Sampling and recruitment

The study focussed upon Libyan healthcare professionals (LHCPs), who provide curative, preventive, promotional or rehabilitative healthcare services to individuals, families or communities [19, 20], and Libyan community leaders (LCLs) who take responsibility for the well-being and improvement of their communities, as well as interacting and communicating with the media and the police as a representative of that particular community [21] as key informants for the individual interviews. The interviewees selected for participating in the interviews met the following criteria: currently works as a healthcare professional or a community leader or both; speaks an Arabic language; has been resident in Benghazi for over ten years; has at least five years’ work experience; meets the eligibility criteria for appointments as set out in the internal regulations of the Social People’s Leadership Institution (that is, 35 years of age or older and has achieved an educational qualification(s)).

In the Libyan context, healthcare professionals are considered those who are public healthcare experts [19, 20], and hold a role similar to their counterparts in other parts of the world. The study sought to recruit a range of Libyan healthcare professionals (LHCPs) and Libyan community leaders (LCLs) such as - official leaders at Benghazi Municipal Council, civic leaders at the General Authority of Islamic Affairs and Endowments of Benghazi, connectors and a council of wise men and tribal elders in Benghazi; or public health academics at Benghazi universities.

### Interview schedule

The interview schedule was compiled via a review of interview schedules used in previous studies addressing the obesity epidemic [22–24]. To elicit the interviewees’ perceptions and insights about obesity in Libyan adults, the interview schedule incorporated both open-ended questions and probes, which were tested and developed in a series of pilot interviews prior to the main fieldwork. The interview schedule covered the following topics: knowledge about obesity; influence of age and gender and race on obesity, socio-economic status (SES), unhealthy eating habits, physical activity and sedentary behaviours, neighbourhood environment issues; influences of culture and religion on obesity; the major challenges facing Libyan health care systems obesity prevention and control strategies.

### Ethical considerations

Ethics approval was granted for this qualitative study from the following three relevant bodies: the Institute of Health Research Ethics Committee (IHREC) at the University of Bedfordshire, UK, Omar Al-Mukhtar University affiliated to the Ministry of Higher Education and Scientific Research, Libya and the Regional Health Ministry in Benghazi, Libya. To comply with ethics regulations, all interviewees were reassured about the following five issues of ethical concern: disclosure; well informed; volunteering; competence and consent. The research participants were fully informed about the nature and purpose of the study through providing them with a participant information document and an informed consent form. Written informed consent was obtained from all participants before participating in the interview.

### Interview process

The researchers sought and obtained five letters of permission from the Omar Al-Mukhtar University – HL’s employer. These letters were addressed to the five institutions with which the proposed participants were affiliated. Using standardised wording, the five letters sought the cooperation of the relevant bodies of research to facilitate access to the target participants in each institution. All interviews were conducted in Arabic language because it is the first language for both the interviewees and the main researcher [HL]. Seventeen of the 21 interviews were conducted at institutional locations during normal business hours when the prospective interviewees were at their premises, while four were conducted at the interviewee’s home. Although a choice of venue was offered to participants, they were restricted in their choice due to the curfew enforced by the Libyan government in response to the political instability in Libya, particularly if the interview is conducted late in the day. Another constraint on the interviews was Libyan cultural restrictions against a male researcher interviewing women. To minimise this issue, the main researcher gave female participants the option of holding the interview in presence of their husbands or a Mahram (an unmarriageable kin) at their own home, consistent with conservative cultural norms and Islamic rules. However, all families of the nine women interviewed gave permission to allow the women to talk to HL without their husbands or Mahrams being present. Once all interviews were recorded using a digital voice recorder, the main researcher transcribed them verbatim in Arabic, including the notes that were taken during the course of the interviews. Back translation was employed in the translation process in this qualitative study, which was achieved by allocating three professional bilingual Libyan certified translators. Interviews lasted up to one hour.

### Data analysis

Using QSR NVivo 10.0 (QSR International, Melbourne, Victoria, Australia), qualitative data was analysed using the Framework Analysis approach. This involved the following stages: familiarisation (transcription and reading of data), identifying a thematic framework (inital coding frameworks developed from both a priori and emerging concepts), indexing (applying the thematical framework to the data), charting (creating charts of data using headings from the thematic framework), and mapping and interpretation (searching for patterns, associations, conceptions and explanations aided by visual displays and plots) [25].

#### Familiarisation with the data

HL carried out the interviews and then transcribed all digitally-recorded interviews verbatim and checked the transcripts for accuracy. CP checked a sample of the transcripts for accuracy. The transcripts were read several times to become familiar with their content and to obtain a thorough understanding [25]. This stage was then used to inform the second stage of the analysis.

##### Identifying a thematic framework

The second stage entailed identifying and organising the key themes and sub-themes into a framework from which the data could then be indexed. The primary thematic framework was developed using the following constructs: Socio-economic Status (SES), unhealthy eating habits, physical activities and sedentary lifestyle patterns, and neighbourhood environment factors. A list of key themes and sub-themes was also derived from the emergent themes identified in the familiarisation stage. This list of key themes was modified into a framework by organising the material into a hierarchy of general topics at the top (parent nodes) to more specific topics (sub-themes or sub-nodes or child nodes) [25].

##### Indexing

The aim of this stage was to thoroughly apply the thematic framework to all the data, using numerical codes to identify specific pieces of data, which correspond to different themes. Decision-making in this stage was based on compare and contrast between the initial themes and the refined themes which emerged and evolved following my sustained immersion in the data. Some of the recurrent themes, sub-themes and other issues that emerged in the initial framework (e.g. SES, unhealthy eating habits, physical activities and sedentary lifestyle, and neighbourhood environment) corresponded to and belonged in the main theme. The indexing process was conducted using NVivo v.10. First a system of “parent nodes” was created to represent individual themes and sub-themes. This was the main basis for refinement of the themes in order to more accurately reflect the data and sub-themes that were emerging. The framework was refined in this stage to ensure that the data pertained to theme only and was not duplicated under several themes [25].

##### Charting

Once the coding was completed, relevant shortened quotations or snippets selected from the transcripts were charted in a new framework matrix, which created in Nvivo to achieve this task, separate Excel spreadsheets using the actual words as stated by the interviewees. Hence, designing the charts assisted in extracting, summarising and organising the data relating to a given theme and presenting it in the form of a chart. This facilitated the process of exploring the details, similarities, and differences expressed on a given theme or concept [25].

##### Mapping and interpretation

The thematic charts were transformed into visual models to help understand how the contextual and explanatory factors were perceived by the interviewees to relate to risk and protective factors associated with obesity. These models were incorporated in the presentation of the findings to clarify the relationships between the themes and sub-themes The interpretation of the themes was supported with original quotations selected to highlight the interesting similarities and differences that occurred between the between the perspectives the health care professionals and the community leaders on obesity [25].

## Results

Table 1 presents a breakdown of study interviewees’ socio-demographic and organisational background.

**Table 1:** breakdown of study interviewees’ socio-demographic and organisational background

Interview number	Age	Gender	Occupation	Sub-categories of the interviewees	Organisation key informant recruited from (coded to preserve anonymity)
1	41–50	Female	Diabetologist	3 Physicians	A
2	51–60	Female	Diabetologist		
3	41–50	Male	Diabetologist		
4	31–40	Female	Diabetes Specialist Nurse	3 Nurses Clinical nutritionists	
5	31–40	Female	Diabetes Specialist Nurse		
6	31–40	Female	Diabetes Specialist Nurse		
7	31–40	Female	Dietician	3 Clinical dietitians	
8	31–40	Female	Dietician		
9	31–40	Female	Dietician		
10	41–50	Male	Senior Academic Leader	3 Lecturers of public health	B
11	41–50	Female	Senior Academic Leader		
12	41–50	Male	Academic staff		
13	61–70	Male	Mufti at a house of fatwa	3 Imams (Sheikhs)	C
14	61–70	Male	Imam of a mosque		
15	61–70	Male	Imam of a mosque		
16	51–60	Male	Municipal Council Member	3 Municipal council members	D
17	51–60	Male	Municipal Council Member		
18	41–50	Female	Municipal Council Member		
19	61–70	Male	Tribal leader	3 Tribal leaders	E
20	51–60	Male	Tribal leader		
21	61–70	Male	Tribal leader		

**Fig. 1:**
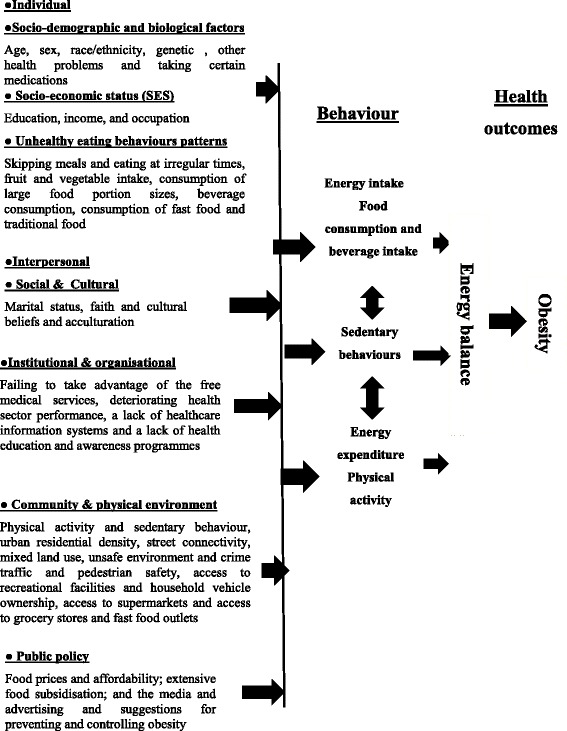
A theoretical model derived from the qualitative research findings

Eleven themes associated with the risk and protective factors of obesity were identified across the 21 interviews conducted. These themes were organised into a theoretical framework (see Fig. 1).

### Factors influencing obesity at the individual level

#### Gender

Analysis of the interviews revealed two distinguishable views concerning gender. One of these, shared by many LHCPs, was that women are more likely than men to be overweight and obese. Their attributions included physiological factors and cultural norms:

*“Women are much more likely than men to become obese in order to attract men as is consistent with our culture.”*

(Female, 36–40, Diabetes Specialist Nurse)

*“Typically, women all over the world are more likely than men to be obese due to physiological causes such as hormones, so I do not think the scenario is any different in Libya, and this what I always see in my work.”*

(Female, 36–40, Dietician)

The second view, predominantly held by the Libyan Community Leaders (LCLs), was that socio-economic status moderated the relationship between gender and obesity:

*“I think poor and uneducated women are more likely to be obese than rich and educated women, while in men obesity occurs in married men more than in singles. Overall, females are more overweight and obese than males.”*

(Male, 46–50, Academic Staff)

#### Occupation

There was consensus across the interviews that the nature of employment offered to Libyans by the Ministry of Labour and Rehabilitation are largely sedentary jobs, as well as the perception that most Libyans are reluctant to engage in manual-labour jobs. The Benghazi Municipal Council members stated that many Libyans were dismissed from their jobs under the Gaddafi regime and were consequently referred to the Human Resources and Unemployment Institution. However, despite not working, Libyans still receive a salary from the government treasury.

*“The old regime sacked many official workers from various vital sectors, such as education, health, economic etc. and the regime transferred them to the Human Resources and Unemployment Institution, despite the government continuing to pay their salaries without anybody carrying out any type of work or service and I think this situation may add some explanations about, why Libyans have become fat people.”*

(Male, 56–60, Municipal Council Member)

#### Unhealthy eating behaviours

##### Skipping meals and eating at irregular mealtimes

The problem of ‘skipping breakfast’ – an unhealthy habit linked to the increased rates of obesity - was raised by the LHCPs groups (although not by the LCLs):.

*“Skipping breakfast is a common unhealthy eating practice of adolescents and adults in Libya because most Libyans stay up late at night. They are night owls and like eating late. They wake up late morning, and take lunch instead of breakfast.”*

(Female, 36–40, Dietician)

*“Based on the views of my patients, many Libyans usually skip breakfast, thinking this to be a healthy practice and a good way of dieting. They then eat a lot at the next meal. They don’t know that skipping breakfast is bad for their health and bodies.”*

(Female, 46–50, Diabetologist)

##### Beverage consumption

There was also consensus across all interviewees that Libyans consume sugary beverages at each meal and between meals throughout the day. However, the tribal leaders held the somewhat naive notion that drinking soft drinks after a heavy meal helps in food digestion. They also held the misconception that no health problems whatsoever result from increasing consumption of sugar-sweetened beverages.

*“No one can deny that consumption of sugary beverages is rising. This harms health and causes increased body-weight. This is really true in Libya as I can see how Libyans are eager to have sugary beverages at each meal, both cold and hot drinks, and at all social events even in the morning. The hot weather is one reason that Libyans like to drink such a quantity of, aside from the fact that all kinds of beverages are cheap and available everywhere.”*

(Female, 36–40, Dietician)

*“We Libyans like to serve more than one kind of sugary beverage at each meal, both cold and hot drinks, and at all social events. Moreover, we consume soft drinks during meals in order to help us to digest the food and help improve our appetite, encouraging us to eat more. I think there are a lot of other health benefits to drinking too many sugary beverage. To me drinking sugary beverage has never caused any health problems; it may even reduce weight instead of increasing it.”*

(Male, 56–60, Tribal leader)

##### Consumption of fast food vs. traditional food

The majority of interviewees perceived that the consumption of fast food has spread widely in Libya and that this is attributable to several factors, which are accessible, affordable, healthy, and delicious food available to the public. The dieticians stated that consumption of take-away and restaurant fast food is not necessarily more dangerous than traditional Libyan foods which they described contain a high concentration of unhealthy fats and carbohydrates. For example:

*“My point is that there is obviously no difference between fast food and home-cooked food, but I think our traditional food is worse than fast food because we use margarine in rice and in all the main meals, so it causes health problems, generally speaking.”*

(Female, 36–40, Dietician)

### Factors influencing obesity at the interpersonal level

#### Faith

Only the Imams that were interviewed raised the role of Islamic beliefs in relation to obesity. All three stated that Islam has given Muslims broad guidelines on how to approach eating and lifestyle:

*“The Shariah Law specifies which foods are halal and which are haram according to the Quran. Islam totally forbids the consumption of alcohol and intoxicants in any amount, also Islam bans the consumption of pork. Islam teaches and advises us to eat and drink in moderation, and to fast, pray, and to engage in physical exercise.”*

(Male, 61–65, Imam of a mosque)

#### Social gatherings

There was consensus across the interviewees that Libyan social functions offer guests a variety of foods in large quantities and, as such, that guests are compelled to over-eat in order to show their respect for their hosts. For example:

*“Social occasions offer traditional food which is oily, and margarine with meat and carbohydrates, which are all unhealthy. And you will be forced to eat a lot to show your respect for the host.”, “I think this behaviour aligned with the old Libyan proverb ‘Eat whatever you are served. If it does not kill you, it will make you obese’.”*

(Female, 36–40, Dietician)

#### Fattening rituals

The tribal leaders raised the practice that occurs in ‘fattening huts’ in which a bride-to-be resides in a confined hut for an extended period of time so that they may rest, drink and eat large quantities of foods over potentially several months as part of her preparation for marriage. This fattening ritual is still practiced by many Libyan tribes, including the Arabs and the Berbers living in the Sahara desert and countryside:

*“Tribes who lives in Libyan villages and in the Sahara, plump women are traditionally seen as more in demand. Our rituals stimulate women before marriage, where women are placed in confinement so they can avoid undoing their effort. They are fed very well with variety of meat and food to prepare them for marriage.”*

(Male, 61–65, Tribal leader)

### Factors influencing obesity at the institutional and organisational level

#### Poor access of free medical services

A view that emerged from the LHCPs was that the Libyan heath system suffers from not having possessing electronic health recording systems. They viewed this as causing problems associated with information management and scheduling which in turn negatively impacts upon service utilisation:

*“…we do not have electronic health record systems. Actually we do not have the patient’s electronic health file to facilitate our job and keep in touch with our patients, notifying them about their schedule and rescheduling their visit.”*

(Female, 56–60, Diabetologist)

The LCLs added that there is a widely held perception among Libyans that the foreign medical staff employed across healthcare services lack experience and competence. They also argued that language barriers between such staff and patients further dissuades Libyans from accessing services:

*“We know our health system’s reliance on foreign workers is a problem. What needs to be considered particularly is the language to communicate with the patients socially, as some foreigners do not speak even the English language. On top of that most of those foreigners and even Libyan staff are unqualified and lack professional experience.”*

(Male, 61–65, Tribal leader)

#### A lack of health education and awareness programmes

There was clear agreement among both LHCPs and LCLs that the Gadaffi regime and the consecutive provisional governments have disregarded health awareness programmes and campaigns. They argued that this has and continues to exacerbate poor health including obesity:

*“No one can hide during the old or new Libyan regime that the performance of the health system is very weak in terms of the provision of curative and preventive services. Look at the health awareness programmes which are completely omitted. I think the absence of health education leads to an increased misconception of simple health norms among Libyans.”*

(Female, 46–50, Diabetologist)

#### Barriers of physical activity

Both groups of interviewees described factors that impeded Libyans from engaging in physical activity. These barriers can be categorised into: Lack of time; religion barriers; cultural barriers; environmental barriers; and politically-related barriers.

#### Lack of time

The LHCPs describes a lack of time due to family and work obligations as a key factor that impedes Libyan people to engage in certain types of physical activities.

*“Despite good living standards for the majority of Libyans, Libyan people always suffer from a lack of time due to family responsibilities, including all the housekeeping (despite the fact that they hire domestic workers) and childcare (despite the fact that they hire babysitters). Others often complain about work commitments or generally being ‘too busy’.”*

(Male, 46–50, Senior Academic Leader)

#### Cultural barriers

Another view raised by the LHCPs was that cultural barriers can include clothing restrictions women cannot be seen in public wearing short sleeves or shorts that deter them from attending public gyms, sport clubs and recreational centres

*“Our culture, however, is still unsatisfied with that, so some strict tribes prohibit women from engaging in physical activities under any condition and consider it a violation of the cultural beliefs.”*

(Male, 46–50, Academic staff)

#### Religion barriers

The LCLs perceived that religion barriers are likely to prevent women rather than men to engage in physical activity among such as Islam obligates women to wear the dress code for Muslim women (Scarf (hijab) and veil (niqab)), as well as the lack of gender-segregated sports facilities.

*“Regardless, our religion gives permission for females to engage in physical activities on condition that Islamic law has imposed that a dress code on the Muslim woman, also gender segregation is maintained, and each gender is provided with trainers.”*

(Male, 61–65, Mufti and a member of a house of fatwa)

#### Environmental barriers

The LCLs perceived that environmental barriers are likely to impede Libyan people to engage in physical activity.

*“Not to mention the weather, Libya’s climate is dominated by an extremely hot during summer and very cold and raining during winter which seems to me as a major barrier that prevents Libyan people to engage in any kind of outdoor physical activity.”*

(Male, 61–65, Tribal leader)

#### Politically-related barriers

There was an explicit acknowledgement among LHCPs and LCLs that Politically-related barriers might play an important role in impeding physical activity amongst Libyan people.

*“I think the current situation in Libya is the crucial cause leading Libyans to stop engaging in any type of outdoor activities due to the destruction of parks, sports and leisure centres, and bicycle lanes, resulting from the war. On top of that, we have an enforced curfew in the evenings by our government.”*

(Male, 51–55, Municipal Council Member)

#### Politically unstable and unsafe environments

Interviewees from both groups perceived that the deteriorated political situation in Benghazi as negatively impacting upon residents’ lifestyle and physical activity. More specifically, they argued that the lack of stability led to unsafe environments and curfews that reduced residents’ motivation and opportunities for physical activity, consequently exacerbating an already-sedentary lifestyle:

*“Actually the Libyan people have a strong desire to live a sedentary lifestyle, aside from the current situation in Benghazi, which seems to me to add insult to injury regarding the already-sedentary lifestyle in Libyans. The unsafe environment and curfew imposed by the government adds fuel to the fire.”*

(Male, 51–55, Municipal Council Member)

*“Even some districts in Benghazi were released from militias. It is still miserable to live in due to lack of basic services such as electricity and energy, and a lack of access to healthy foods due to some supermarkets closing due to the curfew forced by Libyan government”*

(Female, 56–60, Diabetologist)

### Factors influencing obesity at the public-policy level

#### Subsidised food prices

There was agreement across both groups of interviewees that the Libyan government provides extensive subsidies for staple food-commodities including wheat, flour and other miscellaneous commodities which in turn may contribute to over-eating, which tends to promote weight gain.

*“Staple food commodities including wheat, flour, sugar, rice vegetable oils, tea, and miscellaneous are offered to Libyans at very low prices, , where ten loaves of bread costs about 100 Libyan Dirham [five pence]. Personally, I think , despite this subsidy programme benefits Libyan society in certain respects, it is likely to be one of the risk factors fuelling the obesity epidemic in Libya.”*

(Female , 36–40, Dietician)

#### Media and advertising

Interviewees from both groupsو contended that Libyans are exposed intensively to advertising for fast-food restaurants – particularly on television, or via leaflets, flyers, distributed to their homes and on the streets. They argued that all this advertising have an adverse impact on Libyans’ purchasing decisions and promotes unhealthy eating habits.

*“We can see exterior advertising for fast-food restaurants , including any signs, posters, banners. Since we are compelled to stay at home due to the curfew, we can’t deny that we always watch TV and every single hour see a massive variety of advertisements on all channels that encourage us to purchase a variety of food at fast-foods restaurants and supermarkets.”*

(Female, 36–40, Dietician)

## Discussion

As Fig. 1 illustrates, there is a complex relationship between the risk and protective factors identified, and the impact upon lifestyle behaviour, and its consequence upon health outcomes.

One of the main individual-level themes to emerge was the role of gender and the notion that that Libyan women are more vulnerable to obesity than Libyan men. This view aligns with previous studies examining obesity in developing countries [26–28]. As highlighted in the analysis, Libyan women may have fewer opportunities to engage in physical activity due to cultural barriers whereby women cannot be seen in public wearing short sleeves or shorts; or religious barriers whereby Islam obligates women to wear the typical Muslim dress code. Such barriers including the notion that Arab women engaging in physical exercise can be frowned upon has previously been reported [16, 29, 30]. Also identified in the analysis was the cultural phenomenon of fattening rituals; a practice that many Arab women from communities and tribes in North Africa engage in before marriage. This ritual is viewed as a sign of fertility, good health and prosperity [9, 16] but may be playing a contributory role in the Libyan obesity problem.

Another individual-level theme identified was occupation and the narrative from LCLs that many Libyan’s, made unemployment through regime changes, were vulnerable to becoming obese as they received a salary large enough to keep them sedentary and unmotivated to seek employment. This perspective is dissimilar to the findings of several studies conducted in Eastern Mediterranean countries which showed that occupational status is negatively associated with Body Mass Index [31, 32]. This finding underscores the findings reported by the World Health Organization and the Food and Agriculture Organization of the United Nationsthat Libyans generally prefer sedentary or office jobs, and avoid manual labour jobs even though they do not necessarily have the qualifications for an office job [10, 33].

The other individual-level theme to be identified was that of unhealthy eating behaviours. This included the problem of skipping breakfast which LHCPs perceived to be a common habit. The view that this may contribute to the development of obesity is consistent with several epidemiological studies conducted in developing countries, including Arab countries, which have showed objectively demonstrated that the positive association between skipping breakfast and increased body fat [34–36]. Eating at irregular interviews including late at night was also perceived by LCHPs as a common behaviour and likely contributory factor of obesity that, due to poor health literacy, people are generally unaware of. The problem of night-time eating has been previously observed as commonplace in Arab countries of the Middle East and Korea [37] and America [38, 39] and objectively associated with weight-gain.

The sub-group of dieticians interviewed also identified the unhealthy nature of traditional Libyan foods as potentially even more hazardous than fast foods. This view is aligned with previous studies conducted in Arab region which showed that many (local) traditional Arab foods provided by self-catering food outlets or served whether at home or at restaurants or social functions, have a high-energy density, even higher in unhealthy fats, such as margarine and trans fats, sugar and salt than many Western fast foods [11, 40].

There was a misconception about the benefits of sugary drinks among those interviewees who are unspecialised in health matters, such as the tribal leaders, Imams and municipal council members. These interviewees were uncertain as to whether sugary beverages lead to weight-gain or not, but they posited that drinking soft drinks after a heavy meal helps in food digestion. This latter misconception is aligned with Musaiger [16] who found that many people in several Arab countries hold the mistaken concept that cold beverages help to digest junk food. In fact, SSBs can slow digestion and may cause cramping due to diluting the digestive juices [41].

The interviewed Imams also argued that the Islam may protect against obesity through the prohibition of pork and alcohol as well as cultural practices such as fasting during the holy month. Most Imams based their views on citations from the Holy Prophet, quoting lines such as “In movement is a blessing” and pointing out that the Quran persuades people to engage in physical activities. Such views are dissimilar to the findings of previous studies which indicated that Islamic practices are more likely to promote obesity; for example, through restricting women from engaging in physical activities [42].

An interpersonal theme that emerged from the analysis was that of ‘social gatherings.’ As highlighted by several interviewees, there is an expectation in the Libyan culture that people should over-eat to show their respect to the hosts. Although portions tend be large Libyans feel compelled to ‘clean their plate’ as a sign of respect and in order to receive blessing from God since, according to Libyan customs, finishing the food on one’s plate translates into receiving greater blessings from God. These notions align with a study conducted in Iraq which found that rates of obesity were greater among those who eat collectively from one large familial plate as opposed to those who eat from individual plates [43].

Both groups raised the role of Libyan healthcare services. The LHCPs argued that the lack of an electronic health recording (EHR) system negatively impacts upon service utilisation and effectiveness. The findings of a study conducted in Saudi Arabia substantiate this view; they found that a lack of an EHR system was one of the key barriers preventing Saudis from capitalising on the free medical services in Saudi Arabia. Another finding is that the constant short-term contracts for the foreign medical staff which result in a high turnover of staff those expatriates LHCPs who work in shifts in Saudi healthcare facilities [44, 45]. The LCLs centred on the belief that Libyans may be reluctant to take advantage of the free medical services due to negative perceptions over foreign workers’ competence and communication ability. This perception has been identified in previous research conducted in Saudi Araba, which reviewed the historical development and contemporary of the health care system in Saudi Arabia [44, 45]. These studies revealed that the language barriers, which affected by a lack of health care interpretation services, are likely to prevent the healthcare users from capitalising on the free medical services.

The LHCPs argued that Libyans may possess poor health literacy due to an absence of health awareness programmes. The problem of poor health literacy among the general public can be observed by the cultural notion that obesity is a symbol of health and wealth rather than disease and, linked with this view, the acceptance of fattening up rituals. This finding is aligned with those of previous studies conducted in the Arab region that found Arab communities and tribes in North Africa, perceived the belief that plumpness in women is a sign of fertility, prosperity, beauty, health and wealth [9, 16, 46].

A theme particularly relevant to the Benghazi context was that of ‘politically unstable and unsafe environments.’ Interviewees identified how Libyan’s have been vulnerable to the sedentary lifestyles that politically unstable and unsafe environments can produce, given the reduced motivation people hold in engaging with the community, as well as the impact of evening curfews resulting in residents staying at home.Both LHCPs and LCLs agreed that Benghazi is a risky environment to travel through due to the conflicting fighting between the Libyan army and militias which has effectively converted some parts of Benghazi into ‘ghost’ districts. LHCPs felt that, despite most regions having been freed from Islamic State ‘Daesh’ control, the Government-imposed curfew limited residents access to local supermarkets carrying healthy foods and increased reliance on fast-foods.

Both groups of interviewees held broadly similar views about subsidised food, agreeing that the Libyan government subsidies staple food commodities heavily. While this subsidy programme carries certain benefits for Libyan society, it may also be serving to increase the risk of obesity since the foods most heavily subsidised are those which tend to be most unhealthy (given that they produced at a cheaper cost). This notion has been previously argued to contribute to obesity [47, 48] and has been reported in previous research studies carried out in both developed countries [47, 49, 50] and in developing countries [51, 52].

The theme with respect to media and advertising, both groups of interviewees held contended that Libyans are heavily exposed to food-related advertising on TV and in the street, which influence Libyans’ purchasing decisions and promote unhealthy eating habits that fuel the obesity epidemic. Moreover, Libyans tend to be susceptible to propaganda promoting the consumption of sugary drinks and fast foods. There is a significant body of research which reveals that excessive fast-food advertisements are associated with obesity [53, 54].

### Strengths and limitations of the present study

The study is the first in Libya to explore the views of LHCPs and LCLs. However, given the political and security situation in Libya at the time of data collection, the study sample is limited but the findings provide key areas for future research and policy development.The researchers recognise that future research should aim to include the views and experiences of those people who are living with obesity. However, due to the variable political and security environment within Libya, it was not viable to include a sample of obese adults.

### Recommendations

The study findings suggest a range of areas that could be considered at policy and research level:

#### Policy

Participants clearly expressed concern about the efficiency of health services and ability to communicate between patients and practitioners. The Libyan Health Ministry should consider how to improve the health service by creating an electronic health information system that collates the information from all the public and private health organisations, and ensure that the e-health system for all health-service users is up-to-date. In addition, the Libyan health authorities should consider enforcing the use of the Arabic or English language in all healthcare-related communication and provide interpretation services to ensure that all healthcare users can benefit from the free health services provided by the Libyan government. To dispel many of the myths and misconceptions held by Libyans about obesity, such as considering obesity is a symbol of affluence and beauty, the Libyan government should prioritise the development of a comprehensive health education programme. Libyan officials should review the tax rate on unhealthy foods and beverages. The Libyan authorities should improve the availability of affordable healthier foods and beverages in public-service venues.

#### Research

This study focussed only on LHCPs and LCLs and it is important to ascertain the views and experiences of the public too.

## Conclusion

The findings of this study support the view that multifaceted behavioural and societal factors contribute to obesity in Libyan adults. By exploring the risk and protective factors associated with adult obesity among healthcare professionals and Libyan community leaders - this study can contribute to social awareness of the obesity epidemic as well as the social and psychological implications of obesity in adults. It may open the field for others to conduct further research about the social and psychological aspects of obesity. Furthermore, it may provide obese people with support in managing the associated social issues and psychological disorders. One of the unique aspects that this study reveals is the impact of political instability upon sedentary lifestyles – security concerns combined with the daily curfews are discouraging outdoor physical activities. The findings could inform the interventions that are urgently needed for preventing or controlling the obesity epidemic. Such interventions may include reducing the subsidisation of staple but unhealthy food commodities, which fuel the obesity epidemic, and increasing the subsidisation of healthy foods instead. In addition, public health awareness needs to be raised in order to dispel the many myths and misconceptions held by Libyans about obesity. Finally, the Libyan healthcare system needs to be improved in order to encourage Libyans to make use of it.

## Abbreviations

IHREC: Institute of Health Research Ethics Committee
LCLs: Libyan community leaders
LHCPs: Libyan healthcare professionals
SES: Socio-economic status
